# New Insights into the Mechanisms of Pyroptosis and Implications for Diabetic Kidney Disease

**DOI:** 10.3390/ijms21197057

**Published:** 2020-09-25

**Authors:** Jinwen Lin, Ao Cheng, Kai Cheng, Qingwei Deng, Shouzan Zhang, Zehao Lan, Weidong Wang, Jianghua Chen

**Affiliations:** 1 ZJU—UoE Institute, International Campus, Zhejiang University, Haining 314400, China; 11971004@zju.edu.cn; 2 Kidney Disease Center, The First Affiliated Hospital, College of Medicine, Zhejiang University, Hangzhou 310003, China; 3 ZJU—UoE Institute, The University of Edinburgh, 57 George Square, Edinburgh EH8 9JU, UK; 4 Xiangya School of Medicine, Central South University, 172 Tongzipo Road, Changsha 410013, China; 8303180520@csu.edu.cn (A.C.); chengkai@csu.edu.cn (K.C.); 8303180720@csu.edu.cn (Q.D.); 8303181408@csu.edu.cn (S.Z.); 8303191306@csu.edu.cn (Z.L.); 8303191415@csu.edu.cn (W.W.)

**Keywords:** pyroptosis, diabetic, fibrosis, kidney disease, cellular stress

## Abstract

Pyroptosis is one special type of lytic programmed cell death, featured in cell swelling, rupture, secretion of cell contents and remarkable proinflammation effect. In the process of pyroptosis, danger signalling and cellular events are detected by inflammasome, activating caspases and cleaving Gasdermin D (GSDMD), along with the secretion of IL-18 and IL-1β. Pyroptosis can be divided into canonical pathway and non-canonical pathway, and NLRP3 inflammasome is the most important initiator. Diabetic kidney disease (DKD) is one of the most serious microvascular complications in diabetes. Current evidence reported the stimulatory role of hyperglycaemia-induced cellular stress in renal cell pyroptosis, and different signalling pathways have been shown to regulate pyroptosis initiation. Additionally, the inflammation and cellular injury caused by pyroptosis are tightly implicated in DKD progression, aggravating renal fibrosis, glomerular sclerosis and tubular injury. Some registered hypoglycaemia agents exert suppressive activity in pyroptosis regulation pathway. Latest studies also reported some potential approaches to target the pyroptosis pathway, which effectively inhibits renal cell pyroptosis and alleviates DKD in in vivo or in vitro models. Therefore, comprehensively compiling the information associated with pyroptosis regulation in DKD is the main aim of this review, and we try to provide new insights for researchers to dig out more potential therapies of DKD.

## 1. Introduction

According to the International Diabetes Federation Diabetes Atlas, 9th Edition, nearly one billion people are going to get diabetes around the world by 2020, and the data is expected to reach 25% higher by 2030, and 51% higher by 2045 [1]. As one of the most serious microvascular complications of diabetic patients, the incidence rate of DKD is increasing year-by-year as well. Mediated by a variety of inflammatory factors, DKD is a metabolic disease with complex physiological and pathological mechanisms. Various factors such as hyperglycaemia, lipid metabolism disorder, oxidative stress, and advanced glycosylation end products (AGEs) run through the whole progression of DKD. Notably, some of current studies have proved that hyperglycaemia, fatty acids and other damage associated molecules can be identified by some specific pattern recognition receptors (PRRs) in cells, and induce the occurrence of pyroptosis [2], which leads to kidney damage and function decline. We believe that the signal transduction pathways of pyroptosis are inextricably related to the pathogenesis of DKD.

Pyroptosis, a kind of programmed cell death, is induced through an executor protein of cysteine-aspartic proteases 1 (caspase-1) by some immunoactivity cells stimulated by pathogens and danger signals [3]. Pyroptosis has the characteristics from apoptosis and necrosis, including nuclear shrinkage, DNA rupture and staining positive, cell swelling and bursting, and inflammatory reaction. In 1992, Zychlinsky et al. [4] found that *Shigella forsythiae* caused programmed death of infected macrophages, which was considered as apoptosis at that time because of some common characteristics of apoptosis. While a decade passed, in 2001, Cookson et al. [5] first named this caspase-1-dependent apoptosis signalling pathway as “pyroptosis”. The core of pyroptosis is to activate NLRP3 inflammasome, which mediates GSDMD and rapidly causes cell membrane rupture and cell content release, leading to an inflammatory response. The typical manifestations of pyroptosis are increased by expression of intracellular NLRP3 inflammasome and activated caspase-1.

Pyroptosis is a hotspot in bioscience research in recent years, and it has the particularity of natural immune inflammation. It is evident that pyroptosis involves in the study of Alzheimer’s disease [6], Parkinson’s disease [7] and other neurodegenerative diseases, atherosclerosis [8], rheumatoid arthritis [9] and other chronic progressive diseases. As discussed above, some risk factors of DKD, including high glucose (HG), oxidative stress, abnormal lipid metabolism and AGEs, are strongly related with pyroptosis in the occurrence of DKD in present studies.

We estimate the latest studies emerging in the roles of pyroptosis regulation of DKD in the current review, starting from the biology of pyroptosis to obtain an overview of the main mechanism, and then going deep into the pyroptosis regulation in cell stress and NLRP3 inflammasome activation signalling pathways with DKD to lucubrate the association. Subsequently we discuss pyroptosis-mediated renal resident cell death, its pro-inflammatory and pro-fibrogenic propensities in DKD. Through the preceding foreshadowing, we summarize some present therapeutic avenues of DKD through the mechanism of pyroptosis regulation. In simple terms, we try to provide new insights for researchers to extract more potential therapies of DKD.

## 2. Biology of Pyroptosis

Pyroptosis features in cell swelling, rupture, secretion of cell contents and remarkable proinflammation effect, which has been proved to be associated with diverse diseases. According to the different types of caspases and stimulus signals, pyroptosis pathways are divided into canonical pathway (caspase-1) and non-canonical pathway (not caspase-1).

### 2.1. The Canonical Inflammasome Pathway

**Figure 1:**
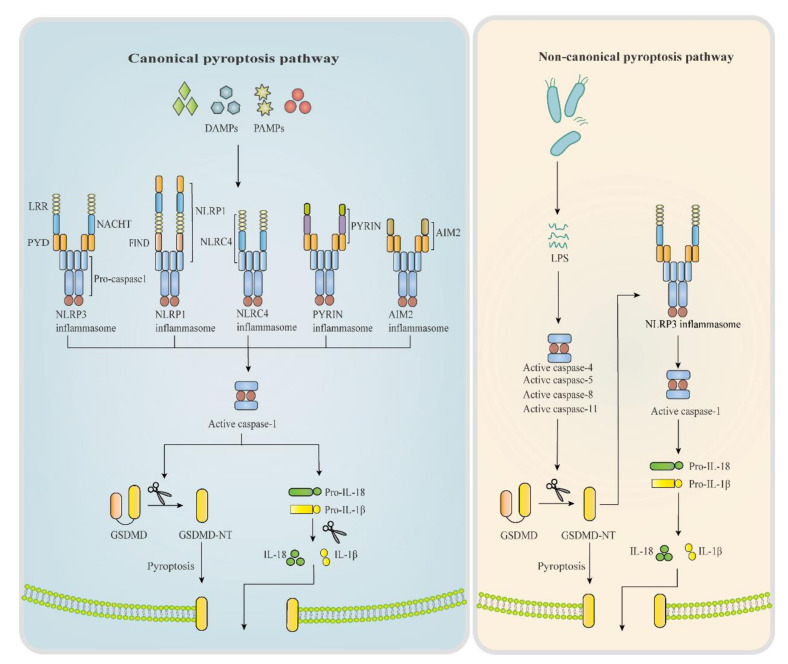
Pyroptosis canonical pathway and non-canonical pathway. Working as the molecular switch of pyroptosis, inflammasomes consist of a sensor PRRs, an adaptor ASC, and an effector pro-caspase-1. During pyroptosis canonical pathway, DAMPs and PAMPs are identified by PRRs, promoting the combination of PRR’s PYD with ASC, thus recruiting pro-caspase-1 and priming complete inflammasome. Activated inflammasome triggers caspase-1 and cleaves GSDMD into GSDMD-NT, which inserts into cell membrane and resulting pore formation, accompanied by the secretion of IL-1β, IL-18. Whereas in non-canonical pathway, it’s caspase-4/5/8/11 that are activated by intracellular LPS or toxins, leading to the same cascaded reactions. PRRs, pattern recognition receptors; ASC, apoptosis-associated speck-like protein; DAMPs, damage-associated molecular patterns; PAMPs, pathogen-associated molecular patterns; GSDMD, gasderminD; LPS, lipopolysaccharides; LRR, leucine rich repeat; PYD, pyrin domain; NACTH, NACTH domain; FIND, Function-to-find domain; GSDMD-NT, GSDMD-N-terminal.

Inflammasomes are multiprotein complexes serving as a molecular switch in pyroptosis and participating in diverse inflammatory diseases. Different types of inflammasomes play the switching role in pyroptosis, mainly including Nod-like receptors (NLRs), pyrin and HIN200 protein absent in melanoma 2 (AIM2) [10]. In particular, NLRs related to pyroptosis contain NLRP1, NLRP3, NLRP6, NLRP7 and NLRC4. Structures of main inflammasomes are shown in Figure 1.

NLRP3 inflammasome proved to be the most connected molecule with pyroptosis occurrence, NLRP3 inflammasome can be activated by diverse stimuli and involves in multiple signalling mechanisms [11,12,13]. Present studies demonstrated its crucial role in pyroptosis initiation and pro-inflammatory cytokines production in DKD [14,15,16]. For instance, NLRP3 deficiency in mice significantly blocks the caspases-1 mediated IL-1β secretion and protects against renal injury in DKD [17,18]. Researchers also observed the obvious upregulation of pyroptosis-related proteins ELAVL1, NLRP3 and caspase-1 in diabetic tissue [19,20]. With deeper researches, many signalling mechanisms of NLRP3 inflammasome activation have been proved to involve in DKD progression [21,22], all interacting with complex metabolic changes in kidneys.

NLRP1/6/7 inflammasome is an NLRP protein family that has similar structures, such as a C-terminal CARD domain and N-terminal pyrin domain, as shown in Figure 1. Studies showed that NLRP6 and NLRP7 activated caspase-1 by interacting with the apoptosis-associated speck-like protein (ASC) in vitro, but NLRP1 processes the function-to-find domain (FIND), which enables the inflammasome to interact with pro-caspase-1 without the ASC domain [23] (Figure 1). A recent study demonstrated the protective effect of NLRP1 in DKD, but the effects of NLRP6 and NLRP7 are unreported [24].

*NLRC4 inflammasome*: NLRC4 belongs to the NLRC protein family, which possesses an N-terminal CARD domain, not the pyrin domain of NLRPs (Figure 1). The CARD domain can recruit pro-caspase-1 and assemble inflammasome. It has been reported that NLRC4 inflammasome is mainly activated by bacterial irritant [25], and the NLRC4-driven IL-1β generation also participates in DKD pathology [26].

*Pyrin inflammasome*: Structurally, there is an N-terminal pyrin domain, two B-box domains, a coil-coiled domain and a C-terminal B30.2 domain in pyrin protein (Figure 1). Pyrin inflammasome is reported to initiate pyroptosis as well, but there is no evidence showing its direct effect on DKD progression [27].

*AIM2 inflammasome*: AIM2 belongs to the HIN200 protein family, possessing a C-terminal pyrin domain and an N-terminal HIN200 domain in structure. It was proved early that double-stranded DNA can be specifically recognized by AIM2 inflammasome [28], and th1e upregulation of AIM2 and mitochondrial DNA (mtDNA) in diabetic patients was observed as well. [29]. However, though some studies have demonstrated the involvement of AIM2 in kidney inflammation, whether it works through the pyroptosis pathway is uncertain [30].

The caspase family serves a function as the bridge protein in pyroptosis. Especially, caspase-1 has been well known to cause pyroptosis and mediate inflammatory response. Caspase-l activation is mediated by inflammasome activation. Subunits of pro-caspase-1 are formed by autolytic hydrolysis of the proenzyme and display as the heterodimer, which further forms into a tetramer, becoming activated caspase-1 [31]. Activated caspase-1 directly cleaves pro-inflammatory cytokines, pro-IL-1β and pro-IL-18 to release IL-1β and IL-18, leading to renal inflammation. In another word, in response to the upstream signalling molecules, activated caspase-l transmits the pyroptosis signal from inflammasomes to the downstream pyroptosis executor protein to release inflammatory cytokines and finally cause pyroptosis.

In the pyroptosis pathway, GSDMD is cleaved by inflammatory caspases at an aspartate site within the linking loop, thereby generating the self-inhibited group GSDMD-C-terminal (GSDMD-CT) and the active group GSDMD-N-terminal (GSDMD-NT) [32,33]. Inhibitory binding between GSDMD-NT and GSDMD-CT leads to the inactivity of GSDMD. In mouse macrophages, GSDMD-NT binds lipids to induce pyroptosis by forming pores of an inner diameter within the membrane. Supporting this prediction, recombinant GSDMD-NT shows robust and specific binding to phosphoinositides and cardiolipin [34]. In the progression of DKD, GSDMD-NT forms pores in the glomerular endothelial cells and gradually triggers cell death, which releases amounts of pro-inflammatory cytokines, such as IL-1β, and activates strong inflammation [35]. Meanwhile, tubular cell injury has something to do with the role of GSDMD-NT in promoting pyroptosis. After transfecting the HG-induced renal tubular epithelial cells (HK-2 cells) with miRNA-452-5p inhibitor, they detected the marked downregulation of the expressions of NLRP3, caspase-1 and GSDMD-NT [32]. However, the concrete effect of GSDMD-NT on cell pore formation in tubular cells has yet to be tested. It is worthy to mention that GSDMD is critical for the secretion of mature IL-1β and IL-18 to extracellular environment, independent of NLRP3 inflammasome and caspase-1 [33], which indicates that it might be insufficient to simply inhibit NLRP3 inflammasome or caspase-1 in pyroptosis pathway. Actually, the molecular mechanisms of GSDMD in pyroptosis have been relatively clear, but novel discoveries of the correlation between pyroptosis and DKD may inspire us to explore more about GSDMD’s effects in kidneys.

### 2.2. The Non-Canonical Inflammasome Pathway

The non-canonical pathway of pyroptosis is mediated by caspase-4 and caspase-5 in humans, and caspase-11 in mice. Caspase-4/5 and caspase-11 have high specificity and affinity, which can be activated by directly binding to lipopolysaccharide (LPS) or lipid A [36]. It was tested that GSDMD was a direct substrate of inflammatory caspases, while activated caspase-1/4/5/11 were tetramers to cleave purified recombinant GSDMD [37]. Recently, caspase-8 has also been reported to show a pyroptotic-like effect in cell death [38] (Figure 1). Caspase 11, in response to LPS, cleaves the 53-kDa inactive precursor form of GSDMD (pro-GSDMD) to generate the pro-pyroptotic N-terminal fragment of mature GSDMD [39], which triggers pyroptosis induction. Meanwhile, it is also proved that GSDMD maturation mediated by caspase-11 contributes to NLRP3-dependent caspase-1 activation and IL-1β release [39]. Activated caspase-4/5/11 can reversely activate NLRP3 inflammasome, driving the maturation and secretion of IL-1β and IL-18. Caspase-11 can even activate non-canonical NLRP3 inflammasome without LPS stimulation [40]. In addition, there is evidence reported that except GSDMD, caspase-4/5/11 can also act on channel protein Pannexin-1 and regulate the release of inflammatory mediators [41].

## 3. Pyroptosis and Cellular Stress in DKD

In DKD, hyperglycaemia induces the overproduction and accumulation of reactive oxygen species (ROS), which initiate oxidative stress. ROS are important activators of NLRP3 inflammasome, contributing to inflammation and cell pyroptosis. Moreover, endoplasmic reticulum (ER) stress can mediate NLRP3 activation and facilitates pyroptosis pathway. These kinds of cellular stresses, which closely involve in pyroptosis stimulation and renal injury, will be discussed below, aiming to provide a more comprehensive insight of pyroptosis pathway in DKD.

### 3.1. Oxidative Stress

**Figure 2:**
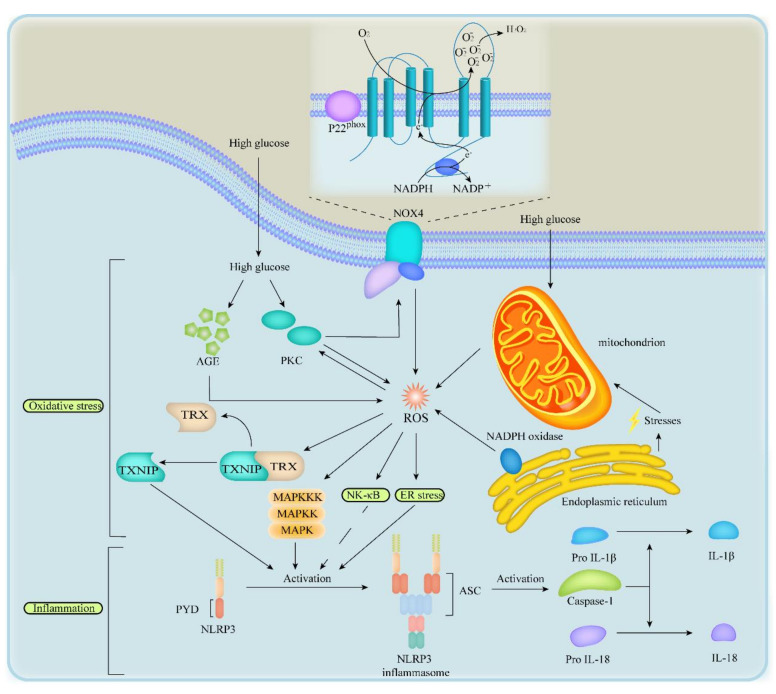
Oxidative stress and ER stress in inflammation in DKD. In oxidative stress, HG activates the overproduction of AGE and PKC, mitochondria damage and NOX4, which promotes the generation of ROS to induce inflammation by the upregulation of TXNIP, p38MAPK signalling pathway and NF-κB signalling pathway. In ER stress, disruption of homeostasis between the ER and mitochondria and NADPH oxidase in ER activates NF-κB signalling pathway by ROS overproduction. In inflammation, pathways above activate NLRP3 inflammasome and caspase-1 and induce secretion of IL-18 and IL-1β. PKC, protein kinase C; AGE, advanced glycosylation end product; NOX4, NADPH oxidases 4; ROS, reactive oxygen species; XNIP, thioredoxin interacting protein; TRX, thioredoxin; NF-κB, nuclear transcription factor; MAPK, mitogen-activated protein kinase.

Oxidative stress is a great promoter in DKD. Lots of diabetic animal models demonstrated that ROS were a major determinant in diabetic kidney disease [42,43]. ROS accumulation could up-regulate the expression of thioredoxin interacting protein (TXNIP), stimulate phosphatidylinositol 3 kinase (PI3K) pathway, p38Mitogen-activated protein kinase (p38MAPK) signalling pathway and nuclear transcription factor (NF-κB) signalling pathway [42,43,44,45,46,47,48,49,50] (Figure 2).

In DKD, NLRP3 upregulation can be caused by activated protein kinase C (PKC), increased polyol pathway flux, overproduced AGEs, increased hexosamine pathway flux and accumulated nicotinamide adenine dinucleotide phosphate oxidases (NOX), which appear to be the most important contributors in the upstream of NLRP3 inflammasome activation (Figure 2). Accumulated ROS give rise to pro-inflammatory responses and cell death including pyroptosis in many ways but specific activation mechanisms are still unknown [42,43,44,45,46,47,48,51].

TXNIP belongs to the thioredoxin system and binds with thioredoxin (TRX), which can resist oxidative stress in resting cells [45]. However, when separated from TRX, TXNIP could activate caspase-1, facilitate IL-1β and IL-18 release, and contribute to pro-inflammatory response and pyroptosis in the kidneys of db/db mice [42,47]. Moreover, it has also been reported that ROS promote the separation of TXNIP and TRX in the upstream of NLRP3 inflammasome activation [47,48]. In general, under oxidative stress, NLRP3 inflammasome is activated by TXNIP and, thus, initiates pyroptosis.

Furthermore, ROS inactivate phosphatase and tensin homolog, leading to PI3K and extracellular signal-regulated kinase (ERK) pathway stimulation in the upstream of pyroptosis. ROS can also activate NLRP3 inflammasome via p38MAPK signalling pathway and NF-κB pathway [46,47,52]. Supporting this prediction, recent studies revealed that NF-κB pathway elicited GSDMD-mediated pyroptosis in DKD mice’s tubular cells. Moreover, activated ROS act on NF-κB pathway, and thus increase the generation of TNF-α, MCP-1 and IL-1β [46,47] (Figure 2). It is worth noting that, in DKD, ROS combine with NO to form peroxynitrites, a potent oxidant and cytotoxic agent, thereby declining the concentration of NO and causing an O2-/NO imbalance, which inhibits the activation of NF-κB pathway and the proliferation of vascular endothelial cells, leading to vascular endothelial injury [53,54,55]. On the other hand, the production of NO declines on account of vascular endothelial injury. These changes form a vicious cycle, leading to a decrease of NO, and constantly contributing to the progression of oxidative stress to some extent. In conclusion, oxidative stress may lead to pyroptosis of some renal resident cells by the activation of ROS to TXNIP pathway and NF-κB pathway.

### 3.2. Endoplasmic Reticulum Stress

ER stress activates NLRP3 inflammasome via a host of effects including the calcium, lipid metabolism, unfolded protein response, and ROS overproduction [56]. In kidney, HG induces ER stress, leading to cells death thereby contributing to glomerular dysfunction in podocytes in DKD [57,58]. Likewise, ROS and hyperglycaemia trigger ER stress in cultured mesangial cells [59]. In turn, the study of a new model demonstrated that the alteration of steady-state communication between mitochondria and ER under ER stress, caused mitochondrial activity dysregulation, accumulation ROS accumulation and NLRP3 inflammasome activation [60]. In rat renal proximal tubule epithelial cells, NLRP3 proteins seem to have a strong correlation with ER stress; activated ER stress can stimulate the NLRP3/caspase-1/IL-1β axis and cause renal inflammation [61].

In addition, the failure of unfolded protein response to alleviate ER stress can induce the overexpression of C/EBP homologous protein (CHOP) which can also activate the NLRP3 inflammasome as a transcriptional factor. In a word, paralleled to the impediment of NLRP3 inflammasome activation, the reduction of ER stress may hold promise as potential avenues for inhibiting pyroptosis. In addition, ER stress primes cells to activate the NF-κB pathway to express pro-IL-1β, thereby promoting the secretion of IL-1β. Although the specific interaction between ER stress and NF-κB pathway is still unknown, activation of NF-κB pathway is exactly paralleled to ER stress (Figure 2). Given its important relationship with DKD and pyroptosis, maybe more experiments should be carried out on ER stress.

## 4. Pyroptosis Regulatory Pathways in DKD

Pyroptosis pathway regulation is very common in diabetic kidneys, and renal injury positively associates with the accumulation of these regulatory molecules. Current studies revealed the critical involvement of NLRP3 inflammasome activation in pyroptosis pathway regulation under DKD condition. Exactly, different pathogen-associated molecular patterns (PAMPs) directly bind with Toll-like receptors (TLRs) and trigger the priming signalling of NLRP3, which stimulates the transcriptional upregulation of NLRP3, caspase-1, pro-IL-1β and pro-IL-18. In this way, inflammasome activation can be fully licensed and prepared for the second step, during which dangerous stimuli are integrated into diverse cellular stress (such as ion fluxes, ROS generation, AGEs accumulation, mitochondria dysfunction, phagosomal destabilization), and play the role of indirect activators. How do these stress stimuli act on pyroptosis indirectly? Here we will introduce several possible signalling pathways, hoping to provide a more complete overview.

### 4.1. TLRs/NF-κB/NLRP3 Inflammasome Signalling Pathway

**Figure 3:**
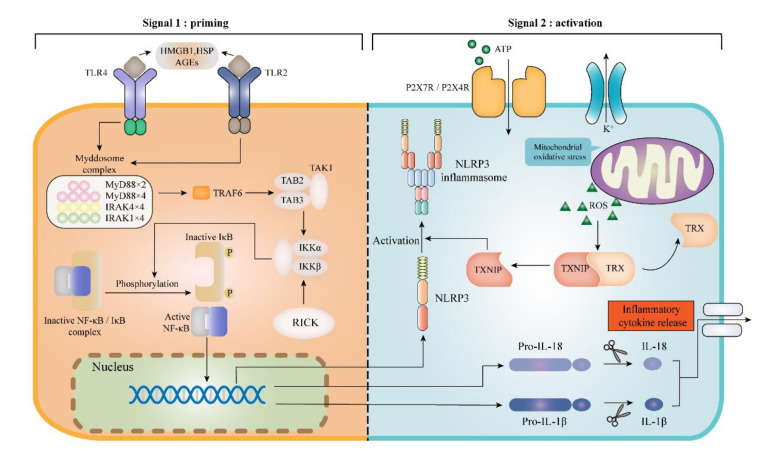
To activate NLRP3 inflammasome needs two signals. The signal 1 (priming; left) is provided by the activation of TLR2 and TLR4 through the stimulation of some DAMPs. Signal 2 (activation; right) is provided by any of numerous PAMPs or DAMPs, such as crystals and eATP. TLR, Toll-like receptor; DAMPs, danger-associated molecular patterns; PAMPs, pathogen-associated molecular patterns; HMGB1, high-mobility group protein box 1; HSP, heat shock protein; AGEs, advanced glycation end products; MyD88, myeloid differentiation factor 88; NF-κB, nuclear factor-κB; TRAF, tumour necrosis factor receptor-associated factor; TAK, transforming growth factor β-activated kinase; IKK, IκB kinase; ROS, reactive oxygen species; TXNIP, thioredoxin interacting protein; TRX, thioredoxin; RICK, Rip-like interactive CLARP kinase; eATP, extracellular ATP.

As an emerging family of receptors, TLRs are important components of pyroptosis regulation molecules, contributing to the pathogenesis of DKD [62]. TLRs have been proved to participate in the assembling of inflammasome and trigger cell pyroptosis. To date, several damage-associated molecular patterns (DAMPs) including high-mobility group protein box 1 (HMGB1) [63], heat shock protein (HSP) [64] and AGEs, have been demonstrated to combine with TLR2 and TLR4, promoting the myeloid differentiation myeloid differentiation factor 88 (MyD88) activation. Afterward, MyD88 interacts with IκB/NF-κB complex to promote the pro-inflammatory cytokines transcription, such as NLRP3, pro-IL-1β and pro-IL-18 (Figure 3). The higher expression of TLR4 and GSDMD is synchronized with renal tubular injury and renal resident cell pyroptosis through the TLR4/NF-κB signalling pathway under the HG circumstances [43]. The expressions of cleaved caspase-1, GSDMD-NT and the secretion of IL-1β and IL-18 are prominently increased in the animal model kidneys of db/db mice. While administration of TAK-242 (a small-molecule inhibitor of TLR4 signalling) and parthenolide (an inhibitor of NF-κB signalling) can suppress the overexpression of these pyroptosis regulatory molecules in a way. Furthermore, a prospective study has shown that the knockdown of TLR4 suppressed the assembling of NLRP3 inflammasome during HG-induced podocyte pyroptosis [65]. In vitro, the expression levels of TLR4, MyD88, NF-κBp65 and NLRP3 protein were detected by Western Blotting in HG-induced glomerular mesangial cells (HBZY-1), and all of them were overexpressed, demonstrating a causative role for TLR4/NF-κB/NLRP3 inflammasome pathway in DKD progression [66].

Notably, besides the cell membrane PRRs, DKD can also be promoted by some cytoplasmic PRRs activation, including NLRC4, NLRC5, and NOD1/2. There is an adaptor protein Rip-like interactive CLARP kinase (RICK, also known as RIP2) interacting with NOD1/2 to assemble a multiprotein signalling platform for the occurrence of cell pyroptosis. In another word, RICK provides a platform for NF-κB to transcript pro-inflammatory expression (including NLRP3, IL-1β and IL-18), so it was named as NOD/RICK/NF-κB signalling pathway [67] (Figure 3). In rat glomerular mesangial cells (RGMCs), NOD1/RICK plays as a sensor to detect cellular stress or hyperglycaemia and lead to NF-κB activation resulting in inflammatory cytokines release, which enhances renal tissue damage [68]. In addition, an experiment, pretreating DKD mice with HG concentration, found NF-κB and c-Jun N-terminal kinase (JNK) were activated in renal tissue and the NLRC4 inflammasome was the upstream activator [26]. Furthermore, NLRC5 was also reported to promote NF-κB and TGF-β (transforming growth factor-β)/Smad (drosophila mothers against decapentaplegic) signalling pathways, eventually triggering pyroptosis and damage to HK-2 cells [69].

### 4.2. TXNIP/NLRP3 Inflammasome Pathway

As introduced before, TXNIP as an important stress senor in vivo, not only connects oxidative stress with inflammasome activation, also interplays with many other pyroptosis regulatory pathways [70]. Actually, it’s been early reported that TXNIP increased progressively with DKD in diabetic kidneys, partly depending on oxidative stress level [71]. Extra TXNIP is able to accelerate the extracellular matrix accumulation and is responsible for tubular oxidative injury [72,73]. Furthermore, the absence of TXNIP protects some types of renal resident cells from HG-induced damage by suppressing mitochondrial glucose metabolism, NADPH oxidase and NOX4 [74]. Considering the great promoting role of TXNIP in mtROS production, above phenomena can be easily understood [75]. Currently, the ROS/TXNIP/NLRP3 biological axis is regarded as a major culprit for pyroptosis initiation in DKD [75]. Wang and his co-workers discovered that in HBZY-1 cells induced by HG, TXNIP, NLRP3, capase-1 and IL-1β were aberrantly overexpressed, furthermore, silencing or knocking out TXNIP gene could suppress the TXNIP/NLRP3 inflammasome pathway, alleviate mesangial cells proliferation, oxidative stress, and extracellular matrix deposition [76]. All these studies uncovered the bridge role of TXNIP between oxidative stress and NLRP3 inflammasome, transmitting danger signalling to NLRP3 and driving downstream responses.

In tubular epithelial cells, the mtROS/TXNIP/NLRP3 axis is observed prominently as well (Figure 3). Overexpressed NLRP3 inflammasome indicates the participation of pyroptosis pathway. Besides, the NADPH oxidase alteration (especially NOX4 isoform) and HG-induction are all underlying factors triggering mtROS/TXNIP/NLRP3 axis in DKD [77,78]. Interestingly, not only can NOX4 stimulate ROS/TXNIP signalling, TXNIP also upregulates the expression of NOX4 through nuclear transcriptional factors NF-κB, Nrf2 and AP-1 [75]. NOX4-derived ROS can activate PKB and ERK pathways, ensuing renal hypertrophy as well as fibrosis in mesangial cells [79]. Taken as a whole, the TXNIP/NLRP3 inflammasome signalling regulates pyroptosis pathway broadly and seems to be a critical contributor to DKD pathogenesis.

### 4.3. ATP/P2X Purinergic Signalling Pathway

ATP/P2X purinergic signalling has become an essential focus in the study of pyroptosis pathway and DKD recently. Former studies testified that hyperglycaemia stimulated ATP secretion in renal resident cells, especially that accumulated extracellular ATP (eATP) can combine with P2X receptors and activate NLRP3 inflammasome in DKD [80,81]. Among different P2X receptors, P2X4R and P2X7R exhibit direct implications in renal cell pyroptosis. P2X7R takes a dual response to eATP and induces the formation of cytoplasmic pores [82]. It has also been reported that P2X7R affects NLRP3 inflammasome activation by promoting NO generation and oxidative stress in high-fat diet mice [83,84]. Essentially, eATP activates NLRP3 inflammasome through P2X7R signalling, resulting in IL-1 production [85,86]. Meanwhile, P2X7 inhibitors relieve renal injury in DKD, further confirming the significant effect of ATP/P2X7 signalling [87]. As to the P2X4R, whose effect on regulating renal sodium transport was underlined in the past [88], latest studies revealed that additional ATP combining with P2X4 could facilitate NLRP3 inflammasome activation in vitro culture HK-2 cells [89]. Consistently, silencing P2X4 gene or applying P2X4 antagonist both suppress NLRP3 expression, with a downregulation of caspase-1, IL-18 and IL-1β levels [89]. Other evidence pointed that activated JAK/STAT signalling pathway could increase P2X4 expression under HG stimulation [90,91].

### 4.4. MAPKs/NLRP3 Inflammasome Signalling Pathway

MAPK signalling pathway is known as a critical regulatory signalling of inflammatory molecules production all the time, and there is lots of evidence reporting its regulatory effect in pyroptosis. MAPK signalling includes three subfamilies, the p38MAPK, ERK and the JNK families.

Vitro studies have indicated that components of p38MAPK signalling pathway can be activated by HG, enhancing NLRP3 inflammasome activation. The eATP and cholesterol crystals [92] are classic NLRP3 inflammasome activators which can trigger the p38MAPKs signalling [93]. An interesting study found that in ASC-knockdown cells, the activation of p38MAPK signalling pathway was reduced and NLRP3 inflammasome expression was downregulated to alleviate DKD as well [94]. Therefore, more research needs to be conducted to reveal the association between NLRP3 inflammasome-mediated p38MAPK signalling and DKD.

Notably, the ERK signalling pathway is also mediated by NLRP3 inflammasome activation. ASC-knockdown cells were pretreated with different TLRs to activate NLRP3 inflammasome. Western blotting results showed that activation of ERK signalling pathway reduced under the circumstance of ASC deficiency. The results indicate that ASC as a NLRP3 inflammasome component can mediate ERK activation through NLRP3 inflammasome activation [94]. During the progression of DKD, oxidative stress, hyperglycaemia and other factors are the upstream stimuli to promote the NLRP3 inflammasome assembling in different renal resident cells. After activating the NLRP3 inflammasome, Raf-1 becomes phosphorylated and causes ERK1/2 activation. Arachidonic acid and eicosanoids are produced by phospholipase A2 (PLA2) phosphorylation under the influence of ERK1/2 then leading to the hyperlipidaemia in renal microenvironment [95]. A previous study demonstrated that spleen tyrosine kinase (Syk) played a vital role in activating NLRP3 to trigger the consequent activation of pro-caspase-1. In DKD rat models induced by streptozotocin (STZ), the higher level of JNK and pyroptosis regulation molecules were detected in HK-2 cells and RGMCs. Administration of Syk-siRNA can inhibit JNK activation under HG concentration. Meanwhile, the NLRP3 inflammasome and IL-1β are significantly down-expressed in HK2 cells and RGMCs [96,97].

## 5. Pathological Role of Pyroptosis in DKD

The pathogenesis of DKD is complex and enormous. Pyroptosis-mediated renal inflammation is an important contributor for DKD, and resident renal cells are key components to trigger and sustain inflammation. Actually, the deterioration of renal function is closely associated with the intensity of pyroptosis regulation molecules. More recent studies showed renal resident cell pyroptosis strongly affects the renal function in DKD progression [35,42,98,99]. Furthermore, various activation signalling pathways of NLRP3 inflammasome contribute to the renal resident cell pyroptosis. In the development of DKD, the unresolved inflammation after sustained kidney injury could promote the fibre formation stage, consequently leading to collagen deposition and accumulation occurring at the later stage, resulting in gradual hardening of renal parenchyma and scar formation, until the kidney function completely fails.

### 5.1. Renal Resident Cell Death

The glomerulus is a highly specialized structure with the glomerular filtration barrier (GFB) comprised of an innermost glomerular endothelial cells (GECs), glomerular basement membrane (GBM) as well as podocytes whereby the slit diaphragm connects interdigitating foot processes. Injury of any GFB components will contribute to albuminuria and glomerular disorder [100]. In DKD, GEC injury and podocyte damage reinforce each other and form a vicious cycle. Additionally, tubular epithelial cell pyroptosis is also the contributor to renal tubule injury.

#### 5.1.1. Glomerular Endothelial Cell Pyroptosis in DKD

In DKD, GECs damage usually occurs early, which has been demonstrated to be induced by HG [98]. GECs, as a part of GFB, are assailable to be damaged by circulating substances, including blood glucose and inflammatory factors. Respectively, the partial colocalization of NLRP3 inflammasome and activated caspase-1 in GECs in histological sections of patients or mice with diabetic, were observed through confocal microscopy. Meanwhile, NLRP3 inflammasome activation contributes to the onset of renal disorder in STZ-treated mice and it is triggered by hyperglycemia in GECs. Of note, their basis of available data shows possibility of NLRP3 inflammasome activation in glomerular cells, which closely relates to the progression of DKD [19].

Previous studies were mainly confined to NLRP3 inflammasome or caspases, but it was limited just to block NLRP3 inflammasome or inflammatory caspases. Thus, some researchers turned their focus to GSDMD and discovered that the release of IL-1β and IL-18 peaked at 24 h in the GECs with HG culture in vitro. Meanwhile, there was an experiment firstly revealing that HG induced GECs pyroptosis targeting GSDMD, and their experiment result demonstrated that silence of GSDMD inhibited GECs pyroptosis [35], indicating the participation of pyroptosis in GECs injury.

#### 5.1.2. Podocyte Pyroptosis in DKD

Podocyte loss in glomerulus is one of the early triggers of DKD. Pyroptosis has been reported as being correlated with the mechanism of podocyte loss [101]. Podocyte pyroptosis was observed in both db/db mice model and STZ-treated mice model, and was testified to be mediated by NLRP3 inflammasome, IL-1β, ROS and other pyroptosis-related molecules. Actually, it is clear that in many pathological conditions, podocytes are one of the priming sources of IL-1β [102]. In addition, activated NLRP3 inflammasome was predominantly upregulated due to NADPH oxidase in podocytes, recruiting a good deal of immune cells and ultimately causing glomerular impairment [103]. Another study of Gao et al. focused on clarifying the role of NLRP3 inflammasome in podocyte damage under HG, through suppressing NLRP3 inflammasome activation. Interestingly, it was found that preventing caspase-1 activation and the IL-1β maturation was the mutual mechanism for both inhibition of ASC/NLRP3 and caspase-1 [104]. Yang et al. reported the NLRP3/ASC/Caspase-1 signalling pathway could be attenuated by blocking of TLR4 during podocyte pyroptosis induced by HG [65]. Previous study found that IL-17A participated in podocyte pyroptosis through ROS/NLRP3/caspase-1 signalling pathway, disrupting the morphological structure of podocytes in vitro by activating the NLRP3 inflammasome and IL-1β expression with the association and involvement of GFB disruption. In contrast, NLRP3 inflammasome inhibition has been shown to prevent podocyte injury. Briefly, podocyte pyroptosis is regulated by different mechanisms in diabetic kidneys and probably results in podocyte loss and aggravates DKD.

#### 5.1.3. Tubular Epithelial Cell Pyroptosis in DKD

Tubular epithelial cell pyroptosis is a risk factor to tubular injury in DKD. As mentioned earlier there is a ROS/TXNIP/NLRP3 inflammasome signalling pathway in tubular epithelial cells which leads to cell pyroptosis. In diabetic patients and db/db mice, the tubular injury and tubular epithelial cell pyroptosis are accompanied by increases of NLRP3 inflammasome, IL-1β and TGF-β expression. Meanwhile, mtROS in tubular epithelial cells are consistently overproduced in db/db mice and may activate the ROS/TXNIP/NLRP3 inflammasome signalling pathway, manifesting itself as a pivotal stimulus to renal tubular injury and renal tubular epithelial cell pyroptosis [42]. It has been highlighted by several evidence that TLRs activation is a trigger of inflammasome formation and cell pyroptosis as well. Notably, in HK-2 cells the suppression of TLR4/NF-κB pathway inhibited pyroptosis, whereby in patients with DKD the upregulation of TLR4 and GSDMD is the leading cause of tubular injury [43]. In addition, in the animal model kidneys of db/db mice, researchers discovered the prominently stimulated expressions of active caspase-1, GSDMD-NT, IL-18 and IL-1β. These alterations were partially amended by the injection of TAK-242 (a small-molecule inhibitor of TLR4 signalling) and parthenolide (an inhibitor of NF-κB signalling).

### 5.2. The Pro-Inflammatory Effect of Pyroptosis

**Figure 4:**
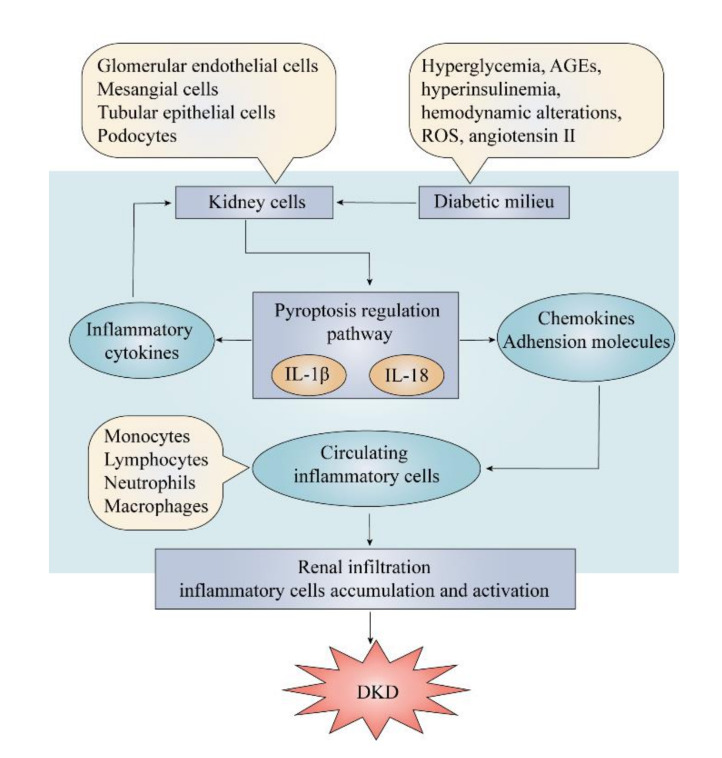
The vicious cycle of pro-inflammatory effect of pyroptosis in DKD. Components of the diabetic milieu act on kidney cells to activate pyroptosis regulation pathway, releasing the pro-inflammatory cytokines, IL-1β and IL-18. They result in renal infiltration by recruiting circulating inflammatory cells through chemokines and adhesion molecules, which amplifies the inflammatory process in the kidney, finally resulting in development and progression of DKD. AGEs, advanced glycosylation end products; ROS, reactive oxygen species; DKD, diabetic kidney disease.

Inflammatory response plays a pivotal role in the development of DKD. IL-1β and IL-18 are two essential pro-inflammatory cytokines synthesized in the pyroptosis regulation which are associated with the expression of chemoattractant cytokines and adhesion molecules in the pathogenesis of inflammatory responses of renal resident cells. Their expression is upregulated in renal resident cells from patients and animals with DKD [105]. These molecules are the core mediators to cause inflammation and renal injury by virtue of their ability to attract monocytes, neutrophils and lymphocytes and other circulating white blood cells to transmigrate into renal tissue. These infiltrating cells can also produce various pro-inflammatory cytokines contributing to the development of renal injury, as well as to deteriorate the inflammatory reaction (Figure 4). IL-1β can stimulates renal cells to produce more pro-inflammatory mediators, such as IL-6, TNF, adhesion molecules intercellular adhesion molecule 1 (ICAM-1), and vascular cell adhesion protein 1 (VCAM-1), and increase the consequent inflammatory response in renal tissue. As early as 1991, Hasegawa et al. found that the expression level of IL-1 was upregulated in macrophage cultured with glomerular basement membranes from diabetic rats [106]. Subsequently, more studies proved that the expression and synthesis of ICAM-1 and VCAM-1 increased after the induction by IL-1 in different types of renal resident cells [107,108]. A study found that the secretion of prostaglandins and PLA2 is stimulated by IL-1β, which indicates the association between IL-1β and hemodynamic abnormalities in glomeruli [109]. Otherwise, IL-18 is also a potent immunoregulatory cytokine that triggers a cascade of inflammatory responses in renal cells [110]. It is reported that application of crocin can inhibit IL-18 expression to attenuate renal injuries under the diabetic milieu. Therefore, IL-1β and IL-18 can induce pro-inflammatory cytokines to increase the inflammatory response and recruit circulating monocytes, macrophages and other lymphocytes, further enhancing local inflammation in renal tissue.

Chemokines and adhesion molecules are important cytokines to facilitate lymphocytes transmigration for worsening the renal inflammation and they can be effectively induced by IL-1β and IL-18 secreted from inflammasomes. C-C motif chemokine 2 (CCL2) secreted from mesangial cells, participates in renal infiltration and kidney damage during the progression of DKD [111]. Otherwise, C-X3-C motif chemokine 1 (CX3CL1) which attracts monocytes, T cells, and natural killer cells, was observed higher expression in the glomerular of diabetic animal models [112]. CCL5 has been proved to correlate with renal interstitial infiltration and renal biopsy samples from DKD patients showed great elevation in renal tubular cells [113]. Except chemokines, adhesion molecules like ICAM-1 and VCAM-1, can upregulate in response to IL-1β and IL-18 and promote the adhesion of lymphocytes and transmigration into renal tissue [114]. A study in diabetes mice has shown increased VCAM-1 expression by endothelial cells, as well as by infiltrating cells in the renal interstitium [115].

As discussed above, increasing evidence has indicated that the pro-inflammatory effect of pyroptosis is a key factor in the pathogenesis of DKD. Disordered metabolism and haemodynamics trigger the upregulation of different inflammatory molecules to establish a chronic inflammation circumstance in diabetic kidney. The chronic inflammation can also act as an upstream event for the further progression of DKD. Therefore, a vicious cycle forms and constantly contributes to renal dysfunction, facilitating the development of DKD (table).

### 5.3. Pro-Fibrogenic Effect

**Figure 5:**
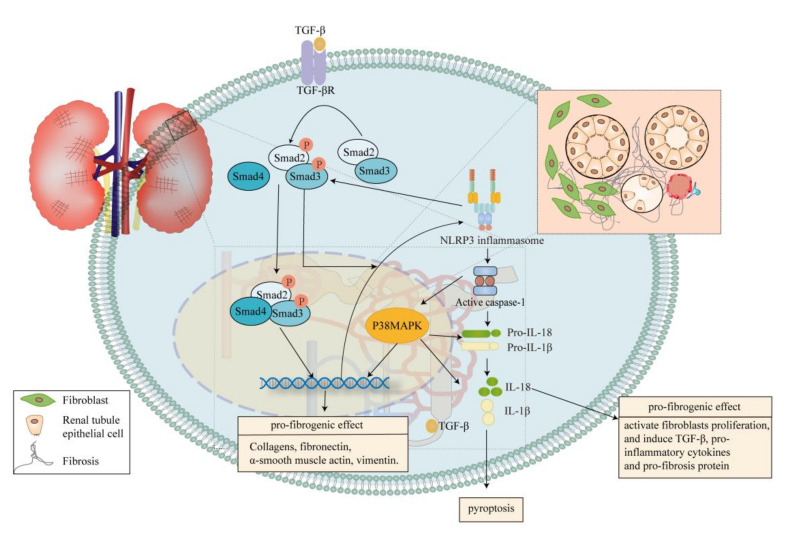
Pyroptosis contributes to interstitial fibrosis. Pyroptosis owing to chronic exposure to diabetic substrates results in cell death, renal inflammation and renal injury. The pathogenic cycle, consisting of the canonical pyroptosis pathway and the TGF-β/Smad signalling pathway, has been proved to stimulate the progress of renal fibrosis. Meanwhile, NLRP3 inflammasome mediates activation of the p38MAPK signalling pathway, regulating the accumulation of fibronectin. Eventually, the crosslink between these pathways increases the production α-SMA, collagen, fibronectin and vimentin, and the accumulation of the ECM, playing a pro-fibrogenic role. Pro-inflammatory cytokines IL-18 from pyroptosis pathway also shows the pro-fibrogenic effect in DKD. Smad, drosophila mothers against decapentaplegic; MAPK, mitogen-activated protein kinase; DKD, diabetic kidney disease; α-SMA, α-smooth muscle actin.

TGF-β/Smad signalling pathway has been reported as an important contributor to renal fibrosis [116]. Likewise, the pro-fibrogenic effect of pyroptosis has been highlighted due to its association with TGF-β/Smad signalling pathway, while activated NLRP3 inflammasome seems to be the bridge between them [117]. In patients with DKD, the production of NLRP3 inflammasome correlates with glomerulosclerosis, interstitial fibrosis and tubular atrophy [118]. TGF-β/Smad signalling pathway can upregulate expression NLRP3, trigger the NLRP3/caspase-1/IL-1β/IL-18 axis and pyroptosis. NLPR3 inflammasome also facilitates the TGF-β/Smad pathway by promoting Smad2/3 phosphorylation which is mediated by TGF-β [117] (Figure 5). This proposal was supported by animal models studies, which indicated that NLRP3 gene knockout greatly improved renal function [17]. Actually, in STZ injection mice model, Ming et al. observed that inhibition of NLRP3 inflammasome took the edge off hypertrophy of glomerular, glomerulosclerosis, expansion of mesangial, interstitial fibrosis, inflammation and expression of TGF-β1, as well as the activation of Smad3. The expression of collagen IV and fibronectin was significantly attenuated with NLRP3 inflammasome inhibition in comparison with the diabetic group. Immunofluorescence staining revealed that inhibition of NLRP3 inflammasome remitted the increasing expression of collagen I. Taken together, these emphasized that the generation of renal extracellular matrix (ECM) as well as interstitial fibrosis in DKD could be effectively suppressed by inhibition of NLRP3 inflammasome [17]. Meanwhile, NLRP3 inflammasome induced by HG suggests prominent association with p38MAPK pathway, due to its involvement in IL-1β and IL-18 production [119], and also regulates the accumulation of fibronectin in basement membrane [120,121] (Figure 5).

The canonical inflammasome pathway of pyroptosis in DKD probably is the critical onset of renal fibrosis by mediating inflammation. However, despite of the interaction between canonical pyroptosis pathway and TGF-β/Smad pathway, it seems that IL-1β and IL-18 also participate in renal fibrosis progression in DKD. Treatment with Canakinumab (one IL-1β inhibitor) also showed great anti-fibrotic effect [122]. In renal biopsies of patients with DKD, IL-18 expression increases in proximal and epithelial tubular cells [123]. In patients with type 2 diabetes, the level of IL-18 in serum and urine is higher than healthy people [124]. Overall, IL-18 gives play to some pro-fibrotic effects by activating fibroblasts proliferation [125], and inducing the synthesis of TGF-β, other pro-inflammatory cytokines and pro-fibrosis protein [126]. These studies further imply that pyroptosis might be a critical driver of renal fibrosis in DKD.

## 6. Therapeutic Drugs Targeting Pyroptosis for DKD Treatment

The current drugs for DKD mainly consist of angiotensin-converting enzyme inhibitors (ACEIs) or angiotensin II receptor blockers (ARBs), which can only delay the progression of DKD rather than reverse it. Therefore, increasing numbers of studies are focusing on the intrinsic renal pathways to discover more potential treatments. Here we will present the latest progress in DKD drug research with a view to pyroptosis pathways, hoping to provide future direction for DKD treatment, and the overview of these renoprotective drugs is shown in Table 1.

### 6.1. Registered Drugs

Although being clinically used, the ACEIs and ARBs are still controversial due to their adverse effects. In comparison, some traditional hypoglycaemic drugs seem to display better and safer effects in DKD treatment, and some of their effects have showed tight connection with renal cell pyroptosis. Typically, sodium-glucose co-transporter type 2 inhibitors, such as dapagliflozin and empagliflozin, could suppress the expression of ASC, caspase-1, IL-1β and the activity of NLRP3 inflammasome in diabetic kidneys [20,127]. MCC950 (one diaryl sulfonylurea) can down-regulate IL-18 and IL-1β expression in renal dendritic cells [128,129], through inhibiting the activation of NLRP3 inflammasome, and prevent inflammation form to further cause renal fibrosis. Dipeptidyl-peptidase-4 inhibitors saxagliptin also suggests similar efficacy [20,130]. Though these drugs have shown significant suppress of pyroptosis key molecules, and alleviated DKD independent of their glucose lowering activity, whether they play these effects through pyroptosis needs to be further confirmed.

### 6.2. Potential Drugs

Though still during trial phase, some potential therapeutic approaches have presented superior pharmacological effects in diabetic kidneys. For instance, the antagonist of adenosine A3 receptor (ADORA3) is proved to attenuate DKD by inhibiting NF-κB/NLRP3 signalling in HK-2, thus blocking the expression of caspase-1, IL-18 and IL-1β [89]. In fact, P2 receptor antagonists such as TNP-ATP and suramin both decreased the expression of pyroptosis related molecules and attenuated DKD, revealing a promising direction [89]. Additionally, short-chain fatty acid NaB is tested to prevent oxidative stress and relieve DKD by suppressing the pyroptosis canonical pathway [35,131].

Notably, with the research trends of epigenetics, the gene therapy of IL-22 and some types of long-non coding RNA (lncRNA) also perform great effects on inhibiting renal cell pyroptosis and, thus, attenuating kidney fibrosis and DKD progression [15,18,32,132,133].

## 7. Conclusions Remarks and Future Perspectives

The study of pyroptosis in DKD is a rich field both in immunology and renal disease, with rapidly emerging insights into the mechanism of pyroptosis regulation in DKD. Inflammasome takes the processes of priming and activation, and mediates the activation of caspase-1, which finally orders the terminal core protein GSDMD to perform its perforation effect. These are all essential links in the process of pyroptosis. Inflammasome is indispensable in pyroptosis and acts as the initiative substance. As a special form of cell death, pyroptosis is like a double-edged sword: On the one hand, moderate cell death is beneficial to protect the kidneys against internal and external dangerous hard plastic damage, whereas, on the other hand, a high amount of renal resident cell pyroptosis leads to a severe inflammatory response, which is the major factor promoting the progression of DKD. An increasing body of research has been devoted to the role of pyroptosis in DKD. However, these studies remain superficial, and how the downstream substances caspase-1 and GSDMD function in the pyroptosis of renal cells remains unclear. On the basis of improving the existing studies’ results, we deepen animal and cell experiments on the activation mechanisms of inflammasome, further revealing some noble studies involved in the pyroptosis regulation and its relationship with DKD, trying to provide a promising direction for the prevention and treatment of DKD. To conclude, there are three new insights for the development of pyroptosis regulation in DKD:

(I) Utilise molecular biological approaches to further explore the concrete mechanisms of downstream substances caspase-1 and GSDMD contributing to DKD. With proved molecular mechanisms we can deepen the understanding of pyroptosis’ role in DKD.
(II) Based on the activation signalling of NLRP3 inflammasome, discover more targeted drugs or small molecules for blocking the pyroptosis regulatory pathway, so as to provide some promising therapies to suppress the progression of DKD.
(III) Focussing more on the pathology of DKD, and deeply discover different upstream stimuli leading to renal resident cell pyroptosis, may provide potential insights for clinical treatment of DKD.

## Figures and Tables

**Table 1:** Therapeutic drugs targeting pyroptosis for DKD treatment.

Types	Drugs	Effects on Renal Tissues	Reference
Registered drugs	SGLT-2i	Suppressing NLRP3 inflammasome activity and the expression of ASC, IL-6, IL-1β, and caspase-1	[20,127]
	Sulfonylureas	Attenuating NLRP3 inflammasome activation	[128,129]
	DPP-4 inhibitors	Reducing the expression of NLRP3and caspase-1, suppressing the release of pro-inflammatory cytokines	[20,130]
Potential drugs	Adenosinergic and Purinergic Receptors Antagonist	Inhibiting the expression of caspase-1, IL-1β and IL-18	[89]
	Short-chain Fatty Acids	Suppressing canonical pyroptosis and oxidative stress	[35,131]
	IL-22 gene therapy	Blocking NLRP3/caspase-1/IL-1β signalling	[18]
	microRNA strategies	Mediating pyroptosis and oxidative stress	[15,32,132,133]

SGLT-2i, Sodium-Glucose Co-Transporter; ASC, apoptosis-associated speck-like protein; DPP-4, Dipeptidyl-peptidase-4.
